# Mechanical circulatory support for cardiogenic shock in takotsubo syndrome

**DOI:** 10.1007/s00392-025-02832-z

**Published:** 2026-02-02

**Authors:** Victoria L. Cammann, Victor Schweiger, Konrad A. Szawan, Davide Di Vece, David Niederseer, Michael Würdinger, Alexander Schönberger, Maximilian Schönberger, Iva Koleva, Julien C. Mercier, Rodolfo Citro, Carmine Vecchione, Eduardo Bossone, Sebastiano Gili, Michael Neuhaus, Jennifer Franke, Benjamin Meder, Miłosz Jaguszewski, Michel Noutsias, Maike Knorr, Thomas Jansen, Fabrizio D’Ascenzo, Wolfgang Dichtl, Dirk von Lewinski, Christof Burgdorf, Behrouz Kherad, Ahmed Elsanhoury, Carsten Tschöpe, Vivian Alice Nelki, Annahita Sarcon, Jerold Shinbane, Lawrence Rajan, Guido Michels, Roman Pfister, Alessandro Cuneo, Claudius Jacobshagen, Mahir Karakas, Wolfgang Koenig, Alexander Pott, Philippe Meyer, Marco Roffi, Adrian Banning, Mathias Wolfrum, Florim Cuculi, Richard Kobza, Thomas A. Fischer, Tuija Vasankari, K. E. Juhani Airaksinen, L. Christian Napp, Rafal Dworakowski, Philip MacCarthy, Christoph Kaiser, Stefan Osswald, Leonarda Galiuto, Christina Chan, Paul Bridgman, Daniel Beug, Stephan B. Felix, Clément Delmas, Olivier Lairez, Ekaterina Gilyarova, Alexandra Shilova, Mikhail Gilyarov, Ibrahim El-Battrawy, Ibrahim Akin, Karolina Poledniková, Petr Toušek, David E. Winchester, Michael Massoomi, Jan Galuszka, Christian Ukena, Gregor Poglajen, Pedro Carrilho-Ferreira, Christian Hauck, Carla Paolini, Claudio Bilato, Yoshio Kobayashi, Ken Kato, Iwao Ishibashi, Toshiharu Himi, Jehangir Din, Ali Al-Shammari, Abhiram Prasad, Charanjit S. Rihal, Kan Liu, P. Christian Schulze, Matteo Bianco, Lucas Jörg, Hans Rickli, Gonçalo Pestana, Thanh H. Nguyen, Michael Böhm, Lars S. Maier, Fausto J. Pinto, Petr Widimský, Ruediger C. Braun-Dullaeus, Wolfgang Rottbauer, Gerd Hasenfuß, Burkert M. Pieske, Heribert Schunkert, Monika Budnik, Grzegorz Opolski, Holger Thiele, Johann Bauersachs, John D. Horowitz, Carlo Di Mario, William Kong, Mayank Dalakoti, Yoichi Imori, Laura Wehling, Norman Mangner, Ulrich Gerk, Thomas Münzel, Filippo Crea, Thomas F. Lüscher, Jeroen J. Bax, Burkhardt Seifert, Jelena R. Ghadri, Christian Templin

**Affiliations:** 1 Department of Cardiology, University Heart Center, University Hospital Zurich, and University of Zurich, Zurich, Switzerland; 2 Internal Medicine B, University Medicine Greifswald, Ferdinand-Sauerbruch-Straße, Greifswald, 17475 Germany; 3 First Clinic of Internal Medicine, Department of Internal Medicine, University of Genoa, Genoa, Italy; 4 Medicine Campus Davos, Davos, Switzerland; 5 Christine Kühne Center for Allergy Research and Education (CK-CARE), Medicine Campus Davos, Davos, Switzerland; 6 Center of Translational and Experimental Cardiology, University Hospital Zurich, Zurich, Switzerland; 7 Heart Department, University Hospital San Giovanni Di Dio E Ruggi d‘Aragona, Salerno, Italy; 8 Department of Vascular Physiopathology, IRCCS Neuromed, Pozzilli, Italy; 9 Department of Medicine, Surgery and Dentistry, University of Salerno, Baronissi, Salerno, Italy; 10 Division of Cardiology, “Antonio Cardarelli” Hospital, Naples, Italy; 11 Centro Cardiologico Monzino, IRCCS, Milan, Italy; 12 Department of Cardiology, Kantonsspital Frauenfeld, Frauenfeld, Switzerland; 13 Department of Cardiology, Heidelberg University Hospital, Heidelberg, Germany; 14 First Department of Cardiology, Medical University of Gdansk, Gdansk, Poland; 15 Department of Internal Medicine III, Division of Cardiology, Angiology and Intensive Medical Care, German Heart Center, University Hospital Halle, Martin-Luther-University Halle-Wittenberg, Halle (Saale), Germany; 16 Department of Cardiology, University Medical Center Mainz, Mainz, Germany; 17 Division of Cardiology, Department of Medical Sciences, AOU Città Della Salute E Della Scienza, University of Turin, Turin, Italy; 18 University Hospital for Internal Medicine III (Cardiology and Angiology), Medical University Innsbruck, Innsbruck, Austria; 19 Division of Cardiology, Medical University of Graz, Graz, Austria; 20 Heart and Vascular Centre Bad Bevensen, Bad Bevensen, Germany; 21 Department of Cardiology, Charité, Campus Rudolf Virchow, Berlin, Germany; 22 Section of Cardiac Electrophysiology, Department of Medicine, University of California-San Francisco, San Francisco, CA USA; 23 Keck School of Medicine, University of Southern California, Los Angeles, CA USA; 24 TJ Health Partners Heart and Vascular, Glasgow, KY USA; 25 Akademisches Lehrkrankenhausder, Klinik Für Akut- Und Notfallmedizin, St.-Antonius-Hospital gGmbH, RWTH Aachen, Eschweiler, Germany; 26 Department of Internal Medicine III, Heart Center University of Cologne, Cologne, Germany; 27 Krankenhaus “Maria Hilf” Medizinische Klinik, Stadtlohn, Germany; 28 Clinic for Cardiology and Pneumology, Georg August University Goettingen, Goettingen, Germany; 29 Vincentius-Diakonissen Hospital, Karlsruhe, Germany; 30 Department of Intensive Care Medicine, University Medical Center Hamburg-Eppendorf, Hamburg, Germany; 31 DZHK (German Centre for Cardiovascular Research), Partner Site Hamburg, Kiel/Luebeck, Hamburg, Germany; 32 Department of Cardiology, Technische Universität München, Deutsches Herzzentrum München, Munich, Germany; 33 DZHK (German Centre for Cardiovascular Research), Partner Site Munich Heart Alliance, Munich, Germany; 34 Medical Center, University of Ulm, Ulm, Germany; 35 Service de Cardiologie, Hôpitaux Universitaires de Genève, Geneva, Switzerland; 36 Department of Cardiology, John Radcliffe Hospital, Oxford University Hospitals, Oxford, UK; 37 Department of Cardiology, Kantonsspital Lucerne, Lucerne, Switzerland; 38 Department of Cardiology, Kantonsspital Winterthur, Winterthur, Switzerland; 39 Heart Center, Turku University Hospital and University of Turku, Turku, Finland; 40 Department of Cardiology and Angiology, Hannover Medical School, Hannover, Germany; 41 Department of Cardiology, King’s College Hospital, London, UK; 42 Department of Cardiology, University Hospital Basel, Basel, Switzerland; 43 Fondazione Policlinico Universitario A. Gemelli IRCCS, Università Cattolica del Sacro Cuore, Rome, Italy; 44 Department of Cardiology, Christchurch Hospital, Christchurch, New Zealand; 45 Department of Cardiology and Cardiac Imaging Center, University Hospital of Rangueil, Toulouse, France; 46 Intensive Coronary Care Unit, Named After N. Pirogov, Moscow City Hospital# 1 , Moscow, Russia; 47 First Department of Medicine, Faculty of Medicine, University Medical Centre Mannheim (UMM) University of Heidelberg, Mannheim, Germany; 48 DZHK (German Center for Cardiovascular Research), Partner Site, Heidelberg-Mannheim, Mannheim, Germany; 49 Third Faculty of Medicine, Charles University in Prague and University Hospital Královské Vinohrady, Prague, Czech Republic; 50 Division of Cardiovascular Medicine, Department of Medicine, College of Medicine, University of Florida, Gainesville, FL USA; 51 Department of Internal Medicine I - Cardiology, University Hospital Olomouc, Olomouc, Czech Republic; 52 Klinik Für Innere Medizin III, Universitätsklinikum Des Saarlandes, HomburgSaar, Germany; 53 Marien Hospital Herne, Ruhr University Bochum, Herne, Germany; 54 Advanced Heart Failure and Transplantation Center, University Medical Center Ljubljana, Ljubljana, Slovenia; 55 Center of Cardiologyof the , Santa Maria University Hospital, CHULN, University of Lisbon, Lisbon School of Medicine, Lisbon Academic Medical Center, Lisbon, Portugal; 56 Klinik Und Poliklinik Für Innere Medizin II, Universitätsklinikum Regensburg, Regensburg, Germany; 57 Local Health Unit N.8, Cardiology Unit, Arzignano, Vicenza, Italy; 58 Department of Cardiovascular Medicine, Chiba University Graduate School of Medicine, Chiba, Japan; 59 Department of Cardiology, Chiba Emergency Medical Center, Chiba, Japan; 60 Division of Cardiology, Kimitsu Central Hospital, Kisarazu, Japan; 61 Dorset Heart Centre, Royal Bournemouth Hospital, Bournemouth, UK; 62 Department of Cardiovascular Diseases, Mayo Clinic, Rochester, MN USA; 63 Division of Cardiology, Heart and Vascular Center, University of Iowa, Iowa City, IA USA; 64 Department of Internal Medicine I, University Hospital Jena, Friedrich-Schiller-University Jena, Jena, Germany; 65 Division of Cardiology, A.O.U San Luigi Gonzaga, Orbassano, Turin, Italy; 66 Department of Cardiology, Kantonsspital St. Gallen, St. Gallen, Switzerland; 67 Department of Cardiology, E.P.E, Centro Hospitalar Universitário de São João, Porto, Portugal; 68 Department of Cardiology, Basil Hetzel Institute, Queen Elizabeth Hospital, University of Adelaide, Adelaide, Australia; 69 Department of Internal Medicine, Cardiology and Angiology, Magdeburg University, Magdeburg, Germany; 70 Berlin Institute of Health (BIH), Berlin, Germany; 71 Department of Cardiology, Medical University of Warsaw, Warsaw, Poland; 72 Department of Internal Medicine/Cardiology, Heart Center Leipzig at Leipzig University, and Leipzig Heart Science, Leipzig, Germany; 73 Structural Interventional Cardiology, Careggi University Hospital, Florence, Italy; 74 Department of Cardiology, National University Heart Centre, Singapore, Singapore; 75 Department of Cardiovascular Medicine, Nippon Medical School, Tokyo, Japan; 76 Department of Internal Medicine and Cardiology, Herzzentrum Dresden, Technische Universität Dresden, Dresden, Germany; 77 Städtisches Klinikum Dresden, Klinik Für Kardiologie, Dresden, Germany; 78 Center for Molecular Cardiology, Schlieren Campus, University of Zurich, Zurich, Switzerland; 79 Royal Brompton and Harefield Hospitals Trust, Imperial College and King’s College, London, UK; 80 Department of Cardiology, Leiden University Medical Centre, Leiden, The Netherlands; 81 Division of Biostatistics, Epidemiology, Biostatistics and Prevention Institute, University of Zurich, Zurich, Switzerland; 82 SwissCardioVascular Clinic (SwissCVC), Private Hospital Bethanien, Zurich, Switzerland

**Keywords:** Takotsubo syndrome, Cardiogenic shock, Mechanical circulatory support, Mortality

## Abstract

**Background:**

Cardiogenic shock complicates takotsubo syndrome (TTS) in approximately 10% of cases. The effectiveness of mechanical circulatory support (MCS) for managing cardiogenic shock in TTS remains unknown.

**Methods:**

We assessed outcomes in TTS patients with cardiogenic shock who received MCS compared to medical therapy only by using data from the International Takotsubo Registry. Two independent propensity scores were computed to investigate outcomes of patients with an intra-aortic balloon pump (IABP) vs. medical therapy only (1:2 propensity score matched cohort) and patients with an Impella vs. medical therapy only (1:1 propensity score matched cohort). The primary endpoint was in-hospital mortality and the secondary outcomes included MCS-related complications.

**Results:**

Among 3740 eligible patients, 309 (8.3%) patients had cardiogenic shock, of whom 112 (36.2%) had MCS and 197 (63.8%) had medical therapy only. After propensity-score matching, the use of an IABP was found to be associated with a lower in-hospital mortality rate than medical therapy only (14.5% vs. 35.5%, *P* = 0.002), while mortality rates in the Impella group and medical therapy only group were comparable (25.0% vs. 29.2%, *P* = 0.75). MCS-related complications occurred in 6.0% of the IABP cohort and in 31.3% of Impella cohort.

**Conclusion:**

Active MCS has been increasingly used for the management of cardiogenic shock in patients with TTS. This observational study could not demonstrate an association with improved mortality with an Impella device, but possibly with an IABP when compared to patients with medical management only. MCS-related complications occurred more frequently in the Impella cohort than in the IABP cohort. Further data are required to confirm results of the present study.

**Graphical Abstract:**

**Figure:**
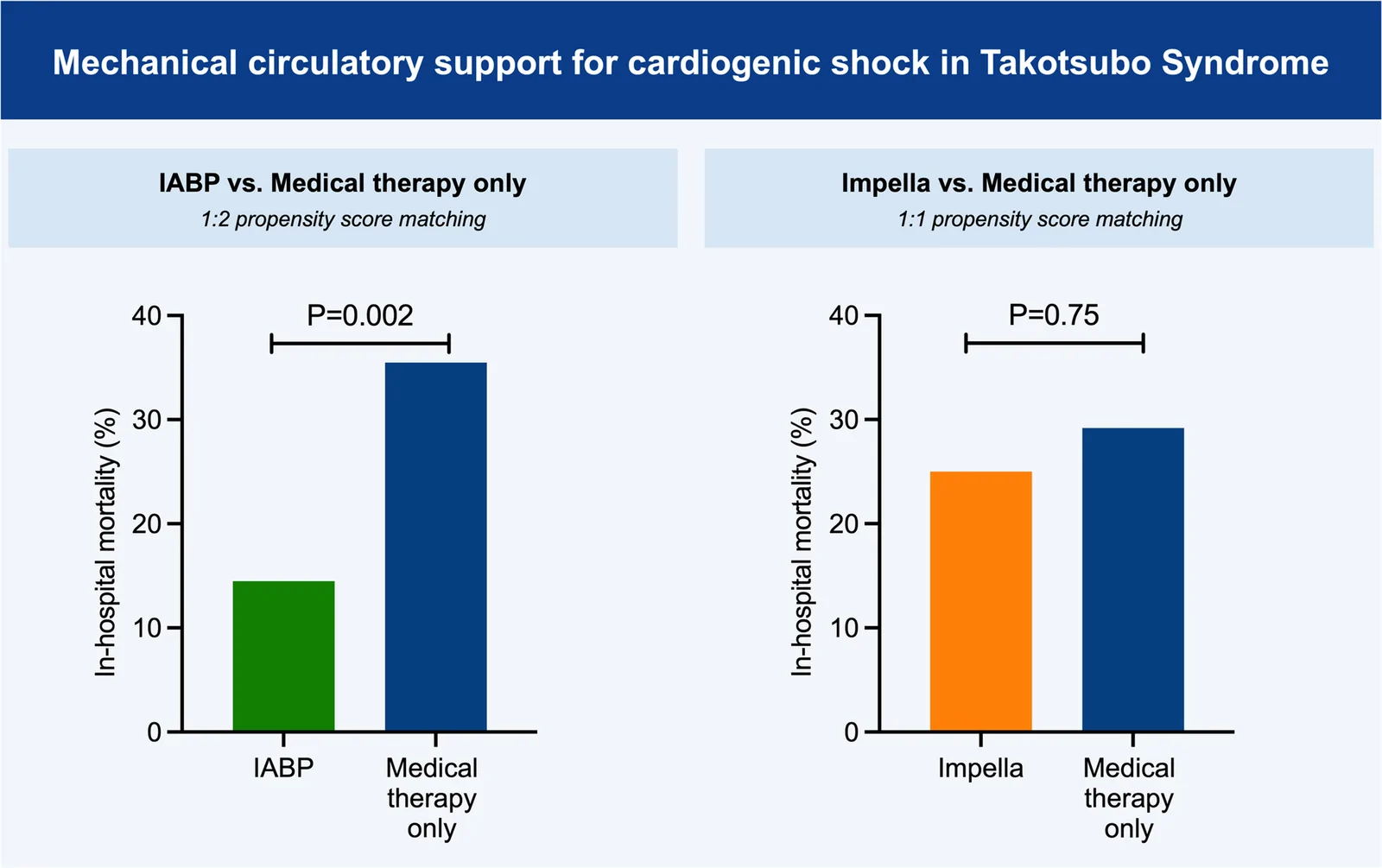

**Supplementary information:**

The online version contains supplementary material available at https://doi.org/10.1007/s00392-025-02832-z.

## Introduction

Takotsubo syndrome (TTS) is an increasingly recognized acute heart failure syndrome characterized by myocardial stunning involving the left ventricle [1]. The incidence rates of severe complications in TTS are comparable to rates observed in acute myocardial infarction [2]. In particular, cardiogenic shock represents a major determinant of acute-phase mortality and occurs in approximately 10% of cases [3]. Poor outcomes in patients with TTS and cardiogenic shock have been reported, with a mortality rate of ranging from 15–30% [3–5]. Mechanical circulatory support (MCS) including intra-aortic balloon pump (IABP), microaxial flow pump (Impella), and extracorporeal membrane oxygenation (ECMO) represents a therapeutic alternative. It may provide greater hemodynamic support than inotropic/vasopressor therapy alone. Current evidence on MCS application in TTS complicated by cardiogenic shock is limited to case reports and small case series without control group suggesting that the use of MCS as a bridge-to-recovery might be associated with improved survival [6–13]. The aim of the present study was to generate first insights into the outcomes and safety of IABP and an Impella device compared to medical therapy alone in TTS patients with cardiogenic shock.

## Methods

### Study design

The International Takotsubo Registry (InterTAK Registry) is an international retrospective and prospective registry including patients with TTS from 56 participating sites in 16 countries [14]. Patients are eligible for inclusion in the registry if the International Takotsubo Diagnostic Criteria (InterTAK Diagnostic Criteria) are met [15]. In the InterTAK Registry, data on demographics, medical history, ECG findings, laboratory values, acute cardiac care measures, and in-hospital complications are captured by central chart review by core team members. Follow-up data are obtained from standardized telephone interviews, chart reviews, or outpatient visits.

The respective local ethics committees or investigational review boards reviewed the study protocol at each collaboration site. Due to the partly retrospective nature of the study, ethics committees of most study centers waived the need for informed consent. At centers where the ethics committees or investigational review boards required informed consent or where patients were included prospectively, formal written consent was obtained from patients or surrogates.

### Study population

For the present study, patients from the InterTAK Registry with TTS complicated by cardiogenic shock were selected. The diagnosis of cardiogenic shock was made by clinical assessment at each collaborating site. Furthermore, shock management strategies were registered. These included medical only, the use of MCS including microaxial left ventricular assist devices (an Impella), IABP, or ECMO, or the use of multiple MCS devices. The shock management strategy was decided at the discretion of the treating physicians at the participating sites. Patients in whom cardiogenic shock was managed without MCS were classified as having received medical therapy only. Patients who received MCS were classified based on the hemodynamic support in IABP, Impella, or EMCO group. The ECMO group includes patients with ECMO support only or in combination with other MCS devices such as IABP (IABP + ECMO) or Impella (ECMELLA).

### Outcomes

The primary outcome was in-hospital mortality. As secondary outcome, device-related complications (limb ischemia, compartment syndrome, retroperitoneal hematoma, arterial thrombosis, aneurysm, minor and major bleeding) were considered as safety endpoints in patients who received MCS. Bleeding events were classified according to Global Use of Strategies To Open Coronary arteries (GUSTO) criteria [16].

### Statistical analyses

Continuous variables are expressed as mean ± standard deviation (SD) or median with interquartile range (IQR) and were tested for differences by using the Student’s *t*-test or Mann Whitney *U*-test, respectively. Categorical variables are summarized as frequencies and percentages and were analyzed using Pearson’s chi-square test or Fisher’s exact test. Multiple group comparisons were conducted with a one-way analysis of variance or the Kruskal–Wallis test.

To assess the association between in-hospital mortality, two independent propensity scores were computed for the use of an IABP or an Impella device. Patients with an IABP were 1:2 matched to patients with medical therapy only and patients with an Impella device were 1:1 matched to patients with medical therapy only. Propensity score matching was performed using a 1:2 ratio for the IABP group and a 1:1 ratio for the Impella group to optimize matching quality and adjust for differences in sample size. The following variables were used for the computation of the propensity scores: age, sex, apical type, InterTAK Classification, [2] physical trigger, left ventricular ejection fraction, and out-of-hospital cardiac arrest (OHCA). These parameters were selected as they may represent potential confounding factors or variables that have been associated with adverse outcomes. OHCA was also included in the propensity score as the most common cause of death after OHCA is irreversible hypoxic brain damage, which is unlikely to be influenced by MCS [17].

Covariate balance before and after propensity score matching was evaluated using standardized mean differences (SMD) and is shown in Love Plots. A sufficient balance after matching was indicated by an SMD threshold of less than 0.1 for all covariates.

A two-sided *P*-value < 0.05 was considered statistically significant. SPSS version 29.0 (IBM Corp., Armonk, NY, USA) and R Version 4.1 (R Foundation for Statistical Computing, Vienna, Austria) were used for statistical analyses, and Prism 8 (GraphPad, La Jolla, CA, USA) for figure design.

## Results

### Study cohort

**Fig. 1:**
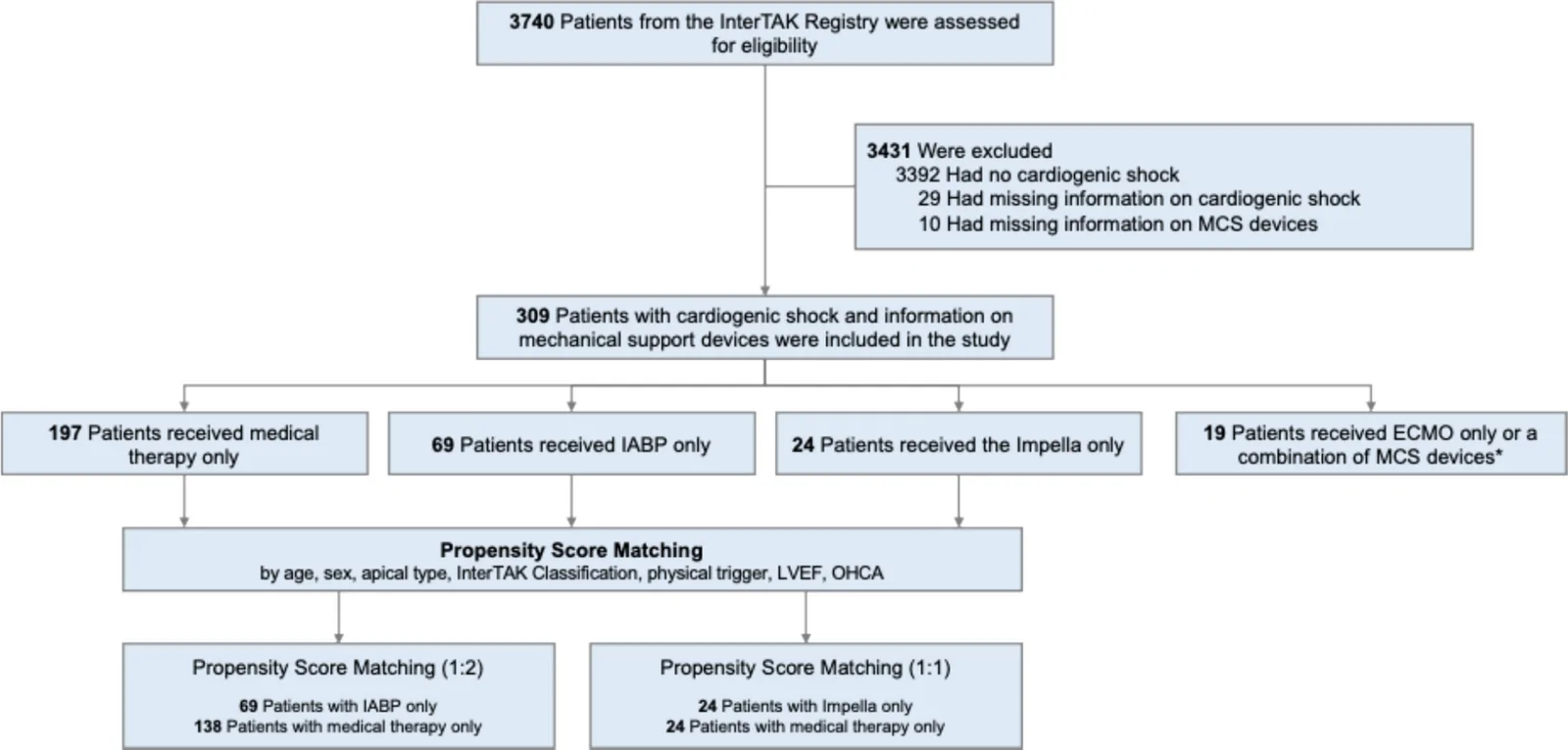
Study population. *Includes 11 patients with ECMO, 5 patients with ECMO and Impella (ECMELLA), and 3 patients with IABP and ECMO. ECMO, extracorporeal membrane oxygenation; IABP, intra-aortic balloon pump; InterTAK Registry International Takotsubo Registry; LVEF, left ventricular ejection fraction; MCS, mechanical circulatory support; OHCA, out-of-hospital cardiac arrest

Of a total of 3740 patients enrolled in the InterTAK Registry, 309 (8.3%) patients with cardiogenic shock were included in the study (Fig. 1). The mean age of the unmatched cohort was 64.6 ± 14.3 years and 69.8% were female. The mean left ventricular ejection fraction was 29.4 ± 11.6%, and the mean left ventricular end-diastolic pressure was 25.1 ± 8.9 mmHg. The percentages of patients who required mechanical ventilation and received inotropic/vasopressor support with or without MCS were 71.8% and 75.6%, respectively. The in-hospital mortality rate was 25.6%. Characteristics of the unmatched cohort are detailed in Table 1.

**Table 1:** Characteristics of TTS patients with cardiogenic shock stratified by treatment regimen

	Total study cohort	Medical Therapy only	IABP only	Impella only	Other MCS°	*P* value
	*N* = 309	*N* = 197	*N* = 69	*N* = 24	*N* = 19	
Demographics						
Female sex—no./total no. (%)	243/309 (69.8)	161/197 (81.7)	52/69 (75.4)	21/24 (87.5)	9/19 (47.4)	0.004
Age—yr—mean ± SD	64.6 ± 14.3 (*N* = 309)	64.6 ± 15.1 (*N* = 197)	64.8 ± 13.7 (*N* = 69)	68.8 ± 9.9 (*N* = 24)	58.5 ± 12.1 (*N* = 19)	0.14
Height—m—mean ± SD	1.63 ± 0.09 (*N* = 216)	1.62 ± 0.09 (*N* = 131)	1.61 ± 0.10 (*N* = 52)	1.66 ± 0.07 (*N* = 21)	1.71 ± 0.13 (*N* = 12)	0.005
BMI—kg/m^2^—mean ± SD	24.6 ± 5.8 (*N* = 12)	24.2 ± 5.7 (*N* = 139)	23.9 ± 4.6 (*N* = 57)	26.6 ± 5.0 (*N* = 21)	28.7 ± 9.6 (*N* = 12)	0.018
Takotsubo Type—no./total no. (%)						
Apical	242/309 (69.5)	146/197 (74.1)	60/69 (87.0)	23/24 (95.8)	13/19 (68.4)	0.015
InterTAK Classification—no./total no. (%)						
Class I: TTS related to emotional stress	29/309 (9.4)	14/197 (7.1)	9/69 (13.0)	4/24 (16.7)	2/19 (10.5)	0.28
Class II: TTS related to physical stress						
Class IIa: TTS secondary to physical activites, medical conditions, or procedures	172/309 (55.7)	113/197 (57.4)	35/69 (50.7)	8/24 (33.3)	16/19 (84.2)	0.007
Class IIb: TTS secondary to acute neurological disorders	39/309 (12.6)	31/197 (15.7)	6/69 (8.7)	2/24 (8.3)	0/19 (0)	0.12
Class III: TTS without an identifiable triggering factor	62/309 (20.1)	35/197 (17.8)	18/69 (26.1)	8/24 (33.3)	1/19 (5.3)	0.06
TTS related to both physical and emotional stress	7/309 (2.3)	4/197 (2.0)	1/69 (1.4)	2/24 (8.3)	0/19 (0)	0.2
Cardiac biomarkers on admission—median (IQR)						
Troponin—factor increase in ULN "	13.86 (2.91–43.83) *N* = 245	10.89 (2.18–37.07) *N* = 159	30.14 (8.40–62.86) *N* = 55	10.74 (1.93–50.07) *N* = 19	7.94 (3.00–17.00) *N* = 12	0.014
Creatine kinase—factor increase in ULN	1.02 (0.60–2.09) *N* = 217	0.96 (0.49–2.06) *N* = 138	1.06 (0.70–1.833) *N* = 48	1.51 (0.67–2.87) *N* = 20	0.98 (0.63–6.53) *N* = 11	0.74
BNP- factor increase in ULN $	8.66 (2.29–38.12) *N* = 146	8.43 (2.13–36.5) *N* = 90	10.05 (2.45–40.41) *N* = 37	8.33 (2.20–53.89) *N* = 18	26.82 (4.04–85.56) *N* = 8	0.69
Inflammatory markers on admission—median (IQR)						
CRP—mg/l	7.00 (1.40–29.80) *N* = 203	7.08 (1.60–35.00) *N* = 127	4.80 (0.48–18.90) *N* = 47	7.04 (2.48–45.08) *N* = 21	9.40 (2.00–70.90) *N* = 11	0.21
WBC—10^3/µl	12.50 (9.80–17.98) *N* = 271	12.05 (9.78–17.22) *N* = 170	13.35 (9.88–20.08) *N* = 66	13.90 (10.50–15.73) *N* = 21	15.22 (9.19–22.50) *N* = 14	0.63
ECG on admission—no./total no. (%)						
Sinus rhythm	234/272 (67.2)	153–175 (87.4)	51/60 (85.0)	19/22 (86.4)	11/15 (73.3)	0.50
ST-segment elevation	136/274 (78.7)	92–177 (52.0)	33/61 (54.1)	4/22 (18.2)	7/14 (50.0)	0.22
ST-segment depression	33/254 (9.5)	23–167 (13.8)	7/52 (13.5)	3/22 (13.6)	0/13 (0)	0.56
T-wave inversion	93/253 (72.7)	61–166 (36.7)	20/52 (38.5)	12/22 (54.5)	0/13 (0)	0.014
QTc—ms	465.7 ± 56.6 (*N* = 217)	466.1 ± 54.7 (*N* = 143)	464.4 ± 63.9 (*N* = 42)	472.6 ± 62.0 (*N* = 22)	450.4 ± 41.6 (*N* = 10)	0.78
Hemodynamics—mean ± SD						
Heart rate—beats/min #	102.0 ± 26.8 (*N* = 239)	98.8 ± 27.2 (*N* = 147)	106.6 ± 21.9 (*N* = 56)	109.9 ± 22.6 (*N* = 23)	104.1 ± 41.6 (*N* = 13)	0.12
Systolic blood pressure—mm Hg #	113.1 ± 32.8 (*N* = 257)	113.3 ± 30.6 (*N* = 156)	113.8 ± 40.0 (*N* = 62)	111.3 ± 25.5 (*N* = 24)	110.2 ± 34.8 (*N* = 15)	0.97
Diastolic blood pressure—mm Hg #	68.3 ± 19.9 (*N* = 246)	69.5 ± 19.5 (*N* = 148)	66.0 ± 23.4 (*N* = 60)	70.9 ± 15.5 (*N* = 23)	62.7 ± 12.6 (*N* = 15)	0.41
Left ventricular ejection fraction—%—‡	29.4 ± 11.6 (*N* = 293)	31.0 ± 10.7 (*N* = 186)	30.2 ± 13.6 (*N* = 65)	18.8 ± 6.6 (*N* = 24)	23.3 ± 8.7 (*N* = 18)	< 0.001
Left ventricular end-diastolic pressure—mm Hg	25.1 ± 8.9 (*N* = 163)	23.8 ± 8.1 (*N* = 108)	25.7 ± 8.6 (*N* = 35)	34.2 ± 9.1 (*N* = 10)	27.1 ± 13.3 (*N* = 10)	0.003
Cardiovascular risk factors/history—no./total no. (%)						
Hypertension	169/306 (48.6)	107/196 (54.6)	36/67 (53.7)	12/24 (50.0)	14/19 (73.7)	0.40
Diabetes mellitus	69/308 (19.8)	43/196 (21.9)	16/69 (23.2)	6/24 (25.0)	4/19 (21.1)	0.98
Current smoking	66/290 (19.0)	43/183 (23.5)	10/65 (15.4)	7/23 (30.4)	6/19 (31.6)	0.61
Hypercholesterolemia	68/301 (19.5)	41/192 (21.4)	15/66 (22.7)	7/24 (29.2)	5/19 (26.3)	0.82
Family history of CAD	31/252 (8.9)	18/156 (11.5)	6/56 (10.7)	6/22 (27.3)	1/18 (5.6)	0.14
Acute cardiac care treatment—no./total no. (%)						
Inotropic agents/vasopressors	263/309 (75.6)	169/197 (85.8)	54/69 (78.3)	21/24 (87.5)	19/19 (100)	0.11
Respiratory therapy	250/309 (71.8)	161/197 (81.7)	25/69 (75.4)	21/24 (87.5)	16/19 (84.2)	0.52
Out-of-hospital cardiac arrest—no./total no. (%)	43/239 (12.4)	31/147 (21.1)	8/50 (16.0)	2/23 (8.7)	2/19 (10.5)	0.37
Support time (days)—median (IQR)	2.00 (1.00–3.00) *N* = 57	-	1.50 (1.00–3.25) *N* = 26	2.00 (1.00–2.00) *N* = 17	3.00 (2.00–7.00) *N* = 14	0.06
In-hospital death—no./total no. (%)	89/309 (25.6)	62/197 (31.5)	10/69 (14.5)	6/24 (25.0)	11/19 (57.9)	0.001

^$^Including upper limits of the normal range for troponin T, high-sensitivity troponin T, and troponin I

^†^Including upper limits of the normal range for brain natriuretic peptide and the N-terminal of prohormone brain natriuretic peptide

^‡^Data obtained during catheterization or echocardiography; if both results were available data from catheterization were used

^#^Data on admission, heart rate and blood pressure may have been influenced by inotropic agents/vasopressors

*BMI* body mass index, *BNP* brain natriuretic peptide, *CRP* c-reactive protein, *CAD* coronary artery disease, *ECG* electrocardiogram, *IQR* interquartile range, *IABP* intra-aortic balloon pump, *MCS* mechanical circulatory support, *QTc* QT interval corrected for heart rate, *TTS* Takotsubo syndrome, *SD* standard deviation, *ULN* upper limit of the normal, *WBC* white blood cell count

°Other MCS includes 11 patients with ECMO, 5 patients with ECMELLA (ECMO + Impella) and 3 patients with IABP + ECMO

### Use of mechanical circulatory support

**Fig. 2:**
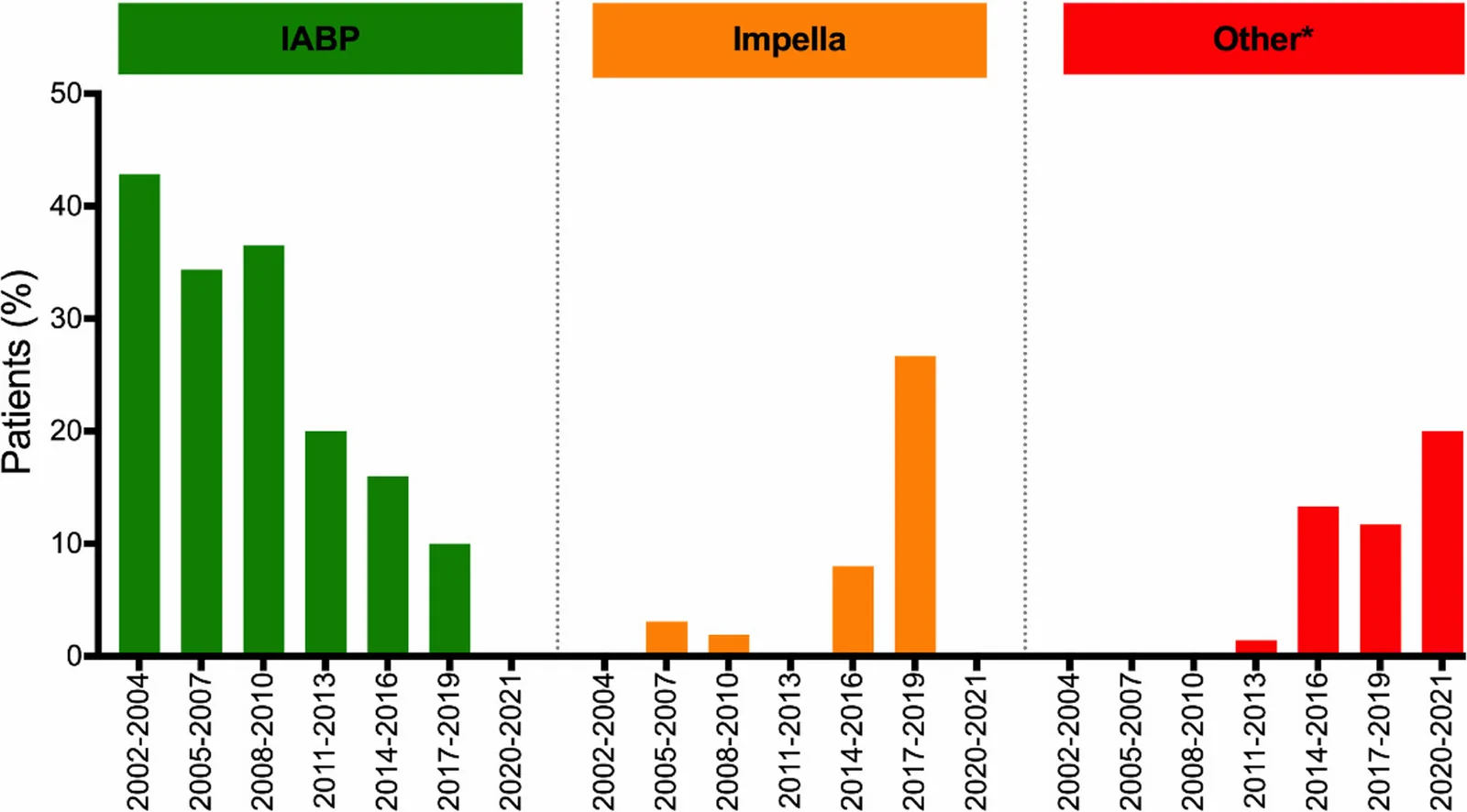
Mechanical circulatory support over time. Usage of mechanical circulatory support (MCS) over time. The use of the IABP has declined, while the Impella and other MCS devices such as the ECMO, ECMELLA or a combination of the IABP and ECMO was more frequently used during the later years of the study. Data for the period from 1999 to 2001 are not presented in the figure as only 1 patient with IABP was included during this period. *Other includes ECMELLA, ECMO, and IABP + ECMO. ECMELLA, Impella + extracorporeal membrane oxygenation; ECMO, extracorporeal membrane oxygenation; IABP, intra-aortic balloon pump

Of the 309 patients with cardiogenic shock, MCS was used in 112 (36.2%) patients while 197 (63.8%) patients received medical therapy only (Table 1, Fig. 1). Among patients who received MCS, 69 (61.6%) had an IABP, 24 (21.4%) had an Impella device (*N* = 3 with Impella 2.5, *N* = 10 with Impella CP, and in 11 the type of Impella was unknown), and 11 (9.8%) had ECMO. Five patients received ECMO in addition to Impella (ECMELLA) and three patients received IABP + ECMO. Over the study period, the utilization of IABP declined after 2010, while active MCS was more frequently used during the last 6 years of enrollment (Fig. 2). Median MCS times were 1.5 days in the IABP group and 2.0 days in the Impella group (Table 1).

### Outcome analysis

After propensity score matching, 69 patients with an IABP were compared to 138 patients with medical therapy only and 24 patients with an Impella device were compared to 24 patients with medical therapy only (Table 2). Median support time was 1.5 (IQR, 1.00–3.25) days in the IABP group and 2.0 (IQR, 1.00–2.00) days in the Impella group. In the matched cohort, the use of IABP was found to be associated with a lower in-hospital mortality rate than medical therapy only (14.5% vs. 35.5%, *P* = 0.002), while in-hospital mortality in the Impella group and medical therapy group were comparable (25.0% vs. 29.2%, *P* = 0.75). Covariate balance before and after the propensity score matching is shown in Supplementary Fig. 1.

**Table 2:** Characteristics of the propensity score matched study cohorts

	1:2 Matched study cohort			1:1 Matched study cohort		
	Medical therapy only	IABP only	*P* value	Medical therapy only	Impella only	*P* value
	*N* = 138	*N* = 69		*N* = 24	*N* = 24	
Demographics						
Female sex—no./total no. (%)	109/138 (79.0)	52/69 (75.4)	0.68	21/24 (87.5)	21/24 (87.5)	1.0
Age—yr—mean ± SD	65.9 ± 14.1 (*N* = 138)	64.8 ± 13.7 (*N* = 69)	0.58	68.0 ± 14.6 (*N* = 24)	68.8 ± 9.9 (*N* = 24)	0.82
Height—m- mean ± SD	1.63 ± 0.09 (*N* = 97)	1.61 ± 0.10 (*N* = 52)	0.30	1.68 ± 0.09 (*N* = 15)	1.66 ± 0.07 (*N* = 21)	0.37
BMI- kg/m^2^—mean ± SD	25.0 ± 6.7 (*N* = 99)	23.9 ± 4.6 (*N* = 57)	0.24	26.2 ± 5.4 (*N* = 15)	26.6 ± 5.0 (*N* = 21)	0.81
Takotsubo Type—no./total no. (%)						
Apical	121/138 (87.7)	60/69 (87.0)	0.88	23/24 (95.8)	23/24 (95.8)	1.0
InterTAK Classification—no./total no. (%)						
Class I: TTS related to emotional stress	13/138 (9.4)	9/69 (13.0)	0.43	4/24 (16.7)	4/24 (16.7)	1.0
Class II: TTS related to physical stress						
Class IIa: TTS secondary to physical activites, medical conditions, or procedures	71/138 (51.4)	35/69 (50.7)	0.92	9/24 (37.5)	8/24 (33.3)	0.76
Class IIb: TTS secondary to acute neurological disorders	18/138 (13.0)	6/69 (8.7)	0.36	2/24 (8.3)	2/24 (8.3)	1.0
Class III: TTS without an identifiable triggering factor	32/138 (23.2)	18/69 (26.1)	0.65	7/24 (29.2)	8/24 (33.3)	1.0
TTS related to both physical and emotional stress	4/138 (2.9)	1/69 (1.4)	0.52	2/24 (8.3)	2/24 (8.3)	0.75
Cardiac biomarkers on admission—median (IQR)						
Troponin—factor increase in ULN "	9.67 (2.00–31.29) *N* = 102	30.14 (8.40–62.86) *N* = 55	0.001	15.15 (3.73–59.34) *N* = 21	10.74 (1.93–50.07) *N* = 19	0.64
Creatine kinase—factor increase in ULN	0.84 (0.49–2.04) *N* = 84	1.06 (0.70–1.83) *N* = 48	0.41	0.92 (0.59–1.68) *N* = 16	1.51 (0.67–2.87) *N* = 20	0.59
BNP—factor increase in ULN $	12.01 (2.68–54.82) *N* = 58	10.05 (2.45–40.41) *N* = 37	0.55	35.73 (2.77–101.2) *N* = 14	8.33 (2.20–53.89) *N* = 18	0.19
Inflammatory markers on admission—median (IQR)						
CRP—mg/l	9.60 (1.65–42.40) *N* = 81	4.80 (0.48–18.90) *N* = 47	0.07	8.90 (1.70–70.90) *N* = 15	7.04 (2.48–45.08) *N* = 21	0.96
WBC—10^3/µl	12.20 (9.49–17.75) *N* = 113	13.35 (9.88–20.08) *N* = 66	0.31	13.72 (10.59–21.36) *N* = 22	13.90 (10.50–15.73) *N* = 21	0.28
ECG on admission—no./total no. (%)						
Sinus rhythm	102/122 (83.6)	51/60 (85.0)	0.81	20/23 (87.0)	19/22 (86.4)	0.95
ST-segment elevation	56/122 (45.9)	33/61 (54.1)	0.30	11/23 (47.8)	4/22 (18.2)	0.035
ST-segment depression	17/119 (14.3)	7/52 (13.5)	0.89	2/23 (8.7)	3/22 (13.6)	0.6
T-wave inversion	40/118 (33.9)	20/52 (38.5)	0.57	8/23 (34.8)	12/22 (54.5)	0.18
QTc—ms	467.9 ± 52.6 (*N* = 95)	464.4 ± 63.9 (*N* = 42)	0.74	472.0 ± 66.3 (*N* = 21)	472.6 ± 62.0 (*N* = 22)	0.98
Hemodynamics—mean ± SD						
Heart rate—beats/min #	98.0 ± 27.8 (*N* = 104)	106.6 ± 21.9 (*N* = 56)	0.048	93.6 ± 16.1 (*N* = 19)	109.9 ± 22.6 (*N* = 23)	0.012
Systolic blood pressure—mm Hg #	113.7 ± 29.5 (*N* = 107)	113.8 ± 40.0 (*N* = 62)	0.98	113.4 ± 29.4 (*N* = 22)	111.3 ± 25.5 (*N* = 24)	0.8
Diastolic blood pressure—mm Hg #	67.6 ± 18.0 (*N* = 104)	66.0 ± 23.4 (*N* = 60)	0.62	65.6 ± 14.4 (*N* = 22)	70.9 ± 15.5 (*N* = 23)	0.24
Left ventricular ejection fraction—%—‡	29.0 ± 9.6 (*N* = 137)	30.2 ± 13.6 (*N* = 65)	0.53	19.4 ± 5.7 (*N* = 24)	18.8 ± 6.6 (*N* = 24)	0.017
Left ventricular end-diastolic pressure—mm Hg	25.1 ± 8.0 (*N* = 78)	25.7 ± 8.6 (*N* = 35)	0.7	24.8 ± 7.9 (*N* = 12)	34.2 ± 9.1 (*N* = 10)	0.74
Cardiovascular risk factors/history—no./total no. (%)						
Hypertension	76/137 (55.5)	36/67 (53.7)	0.81	16/24 (66.7)	12/24 (50.0)	0.24
Diabetes mellitus	34/69 (24.8)	16/69 (23.2)	0.8	8/24 (33.3)	6/24 (25.0)	0.53
Current smoking	34/138 (24.6)	10/65 (15.4)	0.35	4/24 (16.7)	7/23 (30.4)	0.21
Hypercholesterolemia	33/134 (24.6)	15/66 (22.7)	0.78	7/24 (29.2)	7/24 (29.2)	1.0
Family history of CAD	11/109 (10.1)	6/56 (10.7)	0.90	6/22 (27.3)	6/22 (27.3)	1.0
Acute cardiac care treatment—no./total no. (%)						
Inotropic agents/vasopressors	115/138 (83.3)	54/69 (78.3)	0.37	21/24 (87.5)	21/24 (87.5)	1.0
Respiratory therapy	105/138 (76.1)	25/69 (75.4)	0.91	16/24 (66.7)	21/24 (87.5)	0.086
Out-of-hospital cardiac arrest—no./total no. (%)	16/138 (11.6)	8/50 (16.0)	1.0	2/24 (8.3)	2/23 (8.7)	1.0
Support time (days)—median (IQR)	-	1.50 (1.00–3.25) *N* = 26	-	-	2.00 (1.00–2.00) *N* = 17	-
In-hospital death—no./total no. (%)	49/138 (35.5)	10/69 (14.5)	0.002	7/24 (29.2)	6/24 (25.0)	0.75

^$^Including upper limits of the normal range for troponin T, high-sensitivity troponin T, and troponin I

^†^Including upper limits of the normal range for brain natriuretic peptide and the N-terminal of prohormone brain natriuretic peptide

^‡^Data obtained during catheterization or echocardiography; if both results were available data from catheterization were used

^#^Data on admission, heart rate and blood pressure may have been influenced by inotropic agents/vasopressors

*BMI* body mass index, *BNP* brain natriuretic peptide, *CRP* c-reactive protein, *CAD* coronary artery disease, *ECG* electrocardiogram, *IQR* interquartile range, *IABP* intra-aortic balloon pump, *QTc* QT interval corrected for heart rate, *SD* standard deviation, *TTS* Takotsubo syndrome, *ULN* upper limit of the normal, *WBC* white blood cell count

### MCS-related complications

Among the 119 patients who received MCS, data on device-related complications were available for 82 patients. A total of 14 (17.1%) patients had MCS-related complications. MCS-related complications were more frequently observed in patients with the Impella or other MCS devices and were the lowest in patients with IABP (31.3% vs. 37.5% vs. 6.0%, *P* = 0.003). Limb ischemia was the most frequent complication and occurred in 6.0% of patients with IABP, 31.3% of patients with an Impella device, and in 37.5% of those who underwent ECMO only or received a combination of such devices. Major bleeding complications were observed in 1 patient who had hemorrhagic shock after ECMO removal (Table 3).

**Table 3:** Device-related complications

	IABP only	Impella only	Other MCS°	*P* value
	*N* = 69	*N* = 24	*N* = 19	
Complications (total)—no./total no. (%)	3/50 (6.0)	5/16 (31.3)	6/16 (37.5)	0.003
Limb ischemia	1/50 (2.0)	2/16 (12.5)	2/16 (12.5)	-
Compartment syndrome	1/50 (2.0)	-	-	-
Retroperitoneal hematoma	1/50 (2.0)	1/16 (6.3)	-	-
Arterial thrombosis	-	1/16 (6.3)	1/16 (6.3)	-
Aneurysm	-	-	1/16 (6.3)	-
Major bleeding*	-	-	1/16 (6.3)	-
Minor bleeding*	-	1/16 (6.3)	1/16 (6.3)	-

^*^Classified according to GUSTO criteria

°Other MCS includes 11 patients with ECMO, 5 patients with ECMELLA (ECMO + Impella) and 3 patients with IABP + ECMO

## Discussion

In this international registry study, we assessed the outcomes of patients with TTS and cardiogenic shock who were treated with MCS compared to those who received medical therapy alone. MCS devices were used in one-third of cases, with most cases involving the use of IABP or an Impella device. Compared to with medical therapy only, usage of an Impella device was not significantly associated with improved survival, while an IABP was associated with lower mortality when compared to propensity score-matched controls with medical therapy only. The use of MCS was associated with device-related complications in many patients, with the highest rates associated with large-diameter devices.

In the current study, the incidence of cardiogenic shock was nearly 9%, which is consistent with previous studies indicating an incidence ranging from 4 to 20% [18–21]. Contemporary case reports have suggested favorable outcomes when using MCS to manage cardiogenic shock in TTS. Data from a recent meta-analysis including 93 case reports found a pooled survival of nearly 95% and myocardial recovery in all non-fatal cases [6]. However, these positive findings could be subject to publication bias, as reports may disproportionately represent cases with favorable outcomes over those with negative ones.

The pathophysiology of TTS is likely related to a complex multifactorial pathogenesis involving catecholamines and increased sympathetic overactivity leading to microvascular dysfunction [22, 23]. Therefore, inotropic agents should be carefully administered or even avoided. Since the etiology of hypotension/cardiogenic shock in TTS is multifactorial [24, 25], hemodynamic deterioration can be further aggravated by LVOTO, as determined by hypercontractility of the basal segments, and is often associated with moderate-to-severe mitral regurgitation [26–29]. In the acute phase, TTS can be difficult to distinguish from ACS, particularly in the presence of CAD [30, 31]. Transient thrombotic occlusion with spontaneous thrombolysis may result in angiographically normal or near-normal coronary arteries, thereby mimicking TTS. Cardiac MRI has been proposed as a helpful diagnostic tool in this context. The colocalization of myocardial edema and typical wall motion abnormalities in the absence of late gadolinium enhancement has been suggested to provide incremental diagnostic value [32].

Management of cardiogenic shock in TTS patients represents a clinical challenge, especially in the presence of LVOTO, which is estimated to be present in 7–25% of cases and can be further aggravated by inotropes or IABP [33–35]. Therefore, an Impella seems a promising therapeutic concept since left ventricular unloading can reduce filling pressures, LVOTO can be bridged, and coronary microcirculation may be improved [8, 36].

In 2018, investigators from RETAKO Registry demonstrated that LVOTO was the main predictor for hemodynamic deterioration in patients with TTS and cardiogenic shock [37]. In our study, which includes data dating back to 1999, LVOTO was not routinely measured, suggesting that the overall clinical significance of LVOTO was considerably lower at that time. However, the presence or absence of LVOTO may be crucial for device selection in patients with TTS and cardiogenic shock and should be routinely measured.

In the present study, the use of an IABP was associated with improved survival compared to medical therapy alone. IABP augments coronary perfusion during diastole and may also improve microvascular perfusion offering a treatment option for TTS patients with shock and coronary slow flow. Furthermore, one case series suggested that IABP as an adjunctive therapy in patients with TTS and subarachnoid hemorrhage ameliorated cardiogenic shock and cerebral vasospasms [38]. However, IABP may not be a therapeutic option for shock management in the presence of LVOTO, which has been reported in a non-negligible proportion of patients with TTS in association with hemodynamic deterioration. While in our study we did not find a mortality reduction in the Impella group in comparison to matched controls, the presence of physical triggers may have mitigated a potential treatment effect. Patients treated with an Impella presented with more severe hemodynamic compromise and the use of the Impella 2.5 model in some cases may also have influenced outcomes. TTS is an underrated condition and encompasses a heterogeneous population with a wide range of triggering events (e.g., acute neurologic disorders) and especially those with cardiogenic shock had a high acute comorbidity burden. These factors can significantly influence prognosis, thus making it challenging to evaluate the true effectiveness of MCS devices in TTS.

Over the entire study period, the use of IABP declined after 2010, while active MCS devices have increasingly been used. One possible explanation for this trend may be the neutral effect of IABP in AMI-related cardiogenic shock and the corresponding downgrade from a Class IC recommendation to a Class III recommendation in current guidelines for myocardial revascularization [39]. This transition also seems relevant for managing cardiogenic shock in patients with TTS, despite the absence of specific data for TTS and often reliance on acute coronary syndrome protocols. Our study noted a shift from frequent IABP use in early-enrolled TTS patients to an increasing use of an Impella over the years. This period also saw an increase in patients with physical triggers and acute comorbidities, potentially linked to poorer outcomes. The better outcomes with an IABP compared to medical therapy in TTS patients could not be observed with an Impella device, which showed mortality rates similar to medical therapy alone. This change might be in part also attributed to different clinical profiles, with a higher burden of acute comorbidities for TTS cases in recent years, despite adjustments for confounders in our analysis.

TTS patients may rapidly recover and stabilize over the clinical course, which is also reflected by the overall short support time (median, 2 days). Therefore, TTS seems to be a bridgeable disease, and early initiation of short-term MCS as a bridge-to-recovery may be considered. In our study, one-third of patients with MCS had device-related complications, with most complications occurring in the Impella or other MCS groups and the fewest occurring in patients with an IABP. IABP has a smaller catheter size, which might result in fewer complications than large-diameter devices such as the Impella device or ECMO. The incidence of device-related complications in the Impella cohort were similar to those, which have been observed in the DANGER Trial [40]. Early initiation of MCS and early discontinuation of inotropes could potentially lead to improved survival in TTS complicated by cardiogenic shock. Assessment of LVOTO and measurement of LVEDP is crucial before MCS implantation, and device selection may depend on the presence or absence of LVOTO.

## Limitations

The strengths of the study include the use of the largest, international, multicenter cohort of TTS patients with cardiogenic shock, which provided extensive data on patients’ characteristics and prognostic factors. This enabled us to generate propensity score matched analyses based on relevant confounders, comparing TTS controls with medical shock management only to TTS patients managed with either an IABP or an Impella. However, some limitations of our study should be considered. This was a registry-based analysis, and the findings should be interpreted cautiously and regarded as hypothesis-generating. Due to the study's observational nature, causality cannot be established. The definition of shock was not standardized and based on documentation as defined by the investigators at each study site. Therefore, patients with different shock stages (SCAI stage A-E) might have been included [41]. The number of patients in the Impella and other MCS groups was small, and we could not assess the effectiveness of ECMO, ECMELLA, or ECMO combined with IABP. Due to study's real-world and partially retrospective nature, information on LVOTO, dosing and different types of inotropic agents/vasopressors, and no/low-flow time after cardiopulmonary resuscitation was not systematically collected and was not available for this analysis [42]. Furthermore, additional laboratory or hemodynamic parameters beyond those reported were not recorded. Although propensity score matching included relevant confounding factors, residual bias due to unmeasured factors that may influence the outcome cannot be excluded [43]. However, as of today, this study includes the largest available cohort of TTS patients investigating the effectiveness of MCS for managing cardiogenic shock from highly experienced sites.

## Conclusion

In this large, multicenter, observational study, MCS devices were increasingly used to manage cardiogenic shock in TTS. The present study with its limitations could not demonstrate an association with improved mortality with an Impella, but possibly with an IABP when compared matched controls with only medical therapy. The use of MCS was associated with device-related complications in a substantial number of patients. TTS patients are a heterogenous population, and those with cardiogenic shock have a high acute comorbidity burden. Therefore, management of cardiogenic shock in TTS needs to be individually tailored with the selection of MCS based on local expertise to maximize the benefit/risk ratio. Further studies are necessary to verify the present results and to establish a causal relationship.

## Supplementary information

Below is the link to the electronic supplementary material.

ESM 1 (DOCX 536 KB)

## Abbreviations

ECMO: Extracorporeal membrane oxygenation
GUSTO: Global Use of Strategies to Open Occluded Coronary Arteries
IABP: Intra-aortic ballon pump
InterTAK Registry: International Takotsubo Registry
IQR: Interquartile range
LVEDP: Left-ventricular end-diastolic pressure
LVOTO: Left-ventricular outflow tract obstruction
MCS: Mechanical circulatory support
OHCA: Out-of-hospital cardiac arrest
RETAKO: Registry on Takotsubo syndrome
SD: Standard deviation
SMD: Standardized mean difference
TTS: Takotsubo syndrome

## Declarations

Dr. Templin has been supported by a donation from H.H. Sheikh Khalifa bin Hamad Al-Thani to the University of Zurich, Switzerland. Dr. Templin has received institutional grants from Abbott Vascular, Medtronic and SMT as well as consulting grants from Biotronik, Microport and Schnell Medical. Dr. Roffi has received institutional research grants from Boston Scientific, Terumo, Medtronic, Biotronic and Cordis. Dr. Böhm is supported by the Deutsche Forschungsgemeinschaft (German Research Foundation; TTR 219, project number 322900939) and reports personal fees from Abbott, Amgen, Astra Zeneca, Bayer, Boehringer Ingelheim, Cytokinetics, Edwards, Medtronic, Novartis, Servier and Vifor during the conduct of the study. Dr. Lüscher reports outside this work research and educational grants to the institution Abbott, Amgen, AstraZeneca, Boehringer-Ingelheim, Daichi-Sankyo, Menarini Foundation, Novartis, Novo Nordisk, Roche Diagnostics, Sanofi and Vifor and honoraria from Daichi-Sankyo, Menarini, Worksuite Inc., USA. Dr. Niederseer discloses honoraria, consultant fees and/or travel expenses outside this work from Abbott, Amgen, Astra Zeneca, Bayer, Boehringer Ingelheim, Daiichi Sankyo, Dr. Willmar Schwabe GmbH & Co. KG, Emilwood Service Limited, Gerson Lehman Group (GLG) Consulting, Novartis, Novo Nordisk, Pfizer, walk and feel, and Zoll.
