# Supplementary material for: Mechanical circulatory support for cardiogenic shock in takotsubo syndrome
Source: Clin Res Cardiol. 2026 Feb 2;115(11):1944–58. doi: 10.1007/s00392-025-02832-z (PMC13658352; doi:10.1007/s00392-025-02832-z)
Supplement: Supplementary file 1 [file 392_2025_2832_MOESM1_ESM.docx]

**Supplementary Appendix**

# Figure S1. Covariate Balance measured by Standardized Mean Differences.

Covariates balance measured by standardized mean differences (SMD) for medical therapy only vs. IABP (A) and medical therapy only vs. the Impella (B).

Red dots show covariate balance before matching and blue dots after matching.

Grey dashed vertical lines are set at -0.1 and 0.1.

IABP intra-aortic balloon pump; LVEF left ventricular ejection fraction; OHCA out-of-hospital cardiac arrest.
